# Bis[1,3(η^3^)-all­yl][μ-2(η^4^)-1,3-bis­(di­phenyl­phosphino)-2,4-diphenyl­cyclo­buta-1,3-diene-1:3κ^2^ *P*:*P*′]dichlorido-1κ*Cl*,3κ*Cl*-[2(η^5^)-isopropyl­cyclo­penta­dien­yl]-2-cobalt(I)-1,3-dipalladium(II) dichloro­methane solvate

**DOI:** 10.1107/S1600536809018133

**Published:** 2009-05-20

**Authors:** Chin-Pei Chang, Fung-E Hong

**Affiliations:** a Department of Chemistry, National Chung Hsing University, Taichung 402, Taiwan

## Abstract

In the title complex, [CoPd_2_(C_3_H_5_)_2_(C_8_H_11_)Cl_2_(C_40_H_30_P_2_)]·CH_2_Cl_2_, the Co^I^ atom is sandwiched between the cyclo­penta­dienyl and cyclo­butadiene rings. The two diphenyl­phosphine substituents of the cyclo­butadiene ring are situated opposite to each other and bind two Pd^II^ atoms, which are additionally coordinated by a chloride ion and the three C atoms of an allyl ligand, forming a distorted planar coordination environment. The Cl atoms of the dichloro­methane solvent mol­ecule (equal occupancies) and one C atom and its attached H atom of each of the allyl ligands (occupancies 0.55:0.45) are disordered.

## Related literature

For applications of cobalt-containing phosphine-coordinated palladium complexes, see: Chang & Hong (2005 ▶).
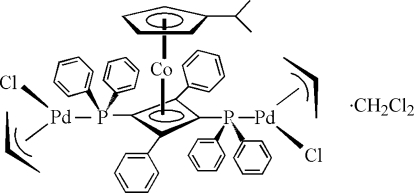


## Experimental

### 

#### Crystal data

[CoPd_2_(C_3_H_5_)_2_(C_8_H_11_)Cl_2_(C_40_H_30_P_2_)]·CH_2_Cl_2_
*M* *_r_* = 1189.44
Triclinic, 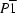

*a* = 10.889 (4) Å
*b* = 12.499 (4) Å
*c* = 21.746 (8) Å
α = 94.617 (7)°
β = 102.136 (7)°
γ = 115.166 (7)°
*V* = 2570.1 (16) Å^3^
*Z* = 2
Mo *K*α radiation
μ = 1.32 mm^−1^
*T* = 298 K
0.25 × 0.20 × 0.15 mm

#### Data collection

Bruker SMART 1000 CCD diffractometer
Absorption correction: multi-scan (*SADABS*; Sheldrick, 1996 ▶) *T*_min_ = 0.734, *T*_max_ = 0.827
14882 measured reflections
10003 independent reflections
6379 reflections with *I* > 2σ(*I*)
*R*_int_ = 0.033

#### Refinement

*R*[*F* ^2^ > 2σ(*F* ^2^)] = 0.047
*wR*(*F* ^2^) = 0.126
*S* = 0.98
10003 reflections
608 parameters
H-atom parameters constrained
Δρ_max_ = 0.60 e Å^−3^
Δρ_min_ = −0.59 e Å^−3^

### 

Data collection: *SMART* (Bruker, 1999 ▶); cell refinement: *SAINT* (Bruker, 1999 ▶); data reduction: *SAINT*; program(s) used to solve structure: *SHELXS97* (Sheldrick, 2008 ▶); program(s) used to refine structure: *SHELXL97* (Sheldrick, 2008 ▶); molecular graphics: *SHELXTL* (Sheldrick, 2008 ▶); software used to prepare material for publication: *SHELXTL*.

## Supplementary Material

Crystal structure: contains datablocks global, I. DOI: 10.1107/S1600536809018133/gk2195sup1.cif

Structure factors: contains datablocks I. DOI: 10.1107/S1600536809018133/gk2195Isup2.hkl

Additional supplementary materials:  crystallographic information; 3D view; checkCIF report

## Figures and Tables

**Table 1:** Selected bond lengths (Å)

Pd1—C45	2.116 (6)
Pd1—C46	2.171 (10)
Pd1—C47	2.195 (6)
Pd1—P1	2.3132 (15)
Pd1—Cl1	2.3901 (17)
Pd2—C48	2.110 (6)
Pd2—C49	2.174 (9)
Pd2—C50	2.223 (6)
Pd2—P2	2.3215 (15)
Pd2—Cl2	2.3897 (18)

**Fig. 1.**
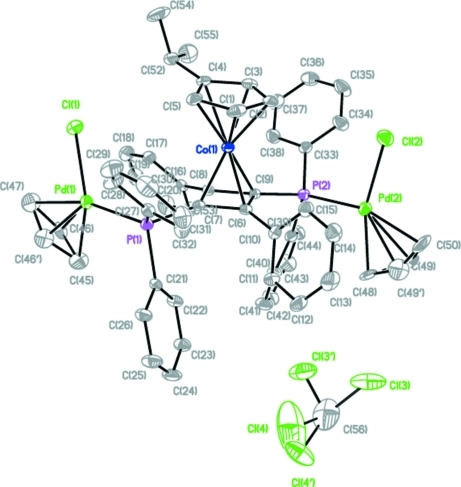
A view of the molecular structure of the title compound with displacement ellipsoids shown at the 40% probability level. Hydrogen atoms have been omitted for clarity.

## supplementary crystallographic information

### Comment

Recently, we have synthesized a series of cobalt-containing phosphine-coordinated palladium complexes which have shown high activities in the Suzuki-Miyaura reaction (Chang *et al.*, 2005). We report herein the synthesis and crystal structure of a cobalt-containing phosphine coordinated palladium complex, a potential catalyst for cross-coupling reactions.

The crystal structure of the title compound reveals that it is a Co^I^ sandwich complex (Figure 1) containing two diphenylphosphino-coordinated palladium moieties attached to the cyclobutadiene ring. The two rings sandwiching the Co atom, cyclopentadienyl and cyclobutadiene, are almost parallel to each other [dihedral angle of 8.46 (8)° ]. The cyclopentadienyl ring bears an isopropyl group as a substituent. The palladium atoms are pentacoordinated; they bind to a chloride ion, a phosphorous atom from the diphenylphosphine ligand and three carbon atoms from the disordered allylic group.

### Experimental

A 100 ml round-bottomed Schlenk flask equipped with a magnetic stirbar and a rubber septum was charged with allylpalladium(II) chloride dimer (0.05 g, 0.15 mmol), (η^5^-C_5_H_4_^i^Pr)Co(η^4^-1,3-Ph_2_-2,4-(PPh_2_)_2_C_4_)(0.11 g, 0.15 mmol) and 10 ml CH_2_Cl_2_. After stirring at room temperature for 1 h, the solvent was removed under reduced pressure. The yellow-colored residue was purified by CTLC (Chromatotron, Harrison model 8924) employed EA/CH_2_Cl_2_=1:1 mixed solvent. The isolated product was identified as the title compound by spectroscopic methods. ^1^H NMR (CDCl_3_, 400 MHz): δ 7.02–8.01 (m, 30H), 5.41 (m, 1H), 4.06 (d, 2H), 2.98 (d, 2H), 0.31 (d, 6H) p.p.m. ^13^C{^1^H} NMR (CDCl_3_, 100 MHz): δ 127.5–132.9, 135.6, 82.0, 80.4, 24.3, 22.0 p.p.m. ^31^P{^1^H} NMR (CDCl_3_, 100 MHz): δ8.6 p.p.m. LRMS: m/s = 1027 [M—Cl]^+^, [C_57_H_59_Cl_2_CoP_2_Pd_2_]. The yield was 53% (0.09 g, 0.08 mmol).

### Refinement

All H atoms were placed in geometrically idealized positions and constrained to ride on their parent atoms with C—H distances in the range 0.93–0.98 Å and *U*_iso_(H) = 1.2*U*_eq_(C). The two allyl groups and the dichloromethane molecule are disordered over two positions with the occupancy factors ratio of 55:45 for the allyl groups and 50:50 for the solvent molecule.

### Crystal data

**Table:**

[CoPd_2_(C_3_H_5_)_2_(C_8_H_11_)Cl_2_(C_40_H_30_P_2_)]·CH_2_Cl_2_	*Z* = 2
*M**_r_* = 1189.44	*F*(000) = 1198
Triclinic, *P*1	*D*_x_ = 1.536 Mg m^−^^3^
Hall symbol: -P 1	Mo *K*α radiation, λ = 0.71073 Å
*a* = 10.889 (4) Å	Cell parameters from 4357 reflections
*b* = 12.499 (4) Å	θ = 2.2–25.4°
*c* = 21.746 (8) Å	µ = 1.32 mm^−^^1^
α = 94.617 (7)°	*T* = 298 K
β = 102.136 (7)°	Parallelepiped, yellow
γ = 115.166 (7)°	0.25 × 0.20 × 0.15 mm
*V* = 2570.1 (16) Å^3^	

### Data collection

**Table:**

Bruker SMART 1000 CCD diffractometer	10003 independent reflections
Radiation source: fine-focus sealed tube	6379 reflections with *I* > 2σ(*I*)
graphite	*R*_int_ = 0.033
Detector resolution: 0 pixels mm^-1^	θ_max_ = 26.1°, θ_min_ = 2.0°
φ and ω scans	*h* = −13→9
Absorption correction: multi-scan (*SADABS*; Sheldrick, 1996)	*k* = −15→14
*T*_min_ = 0.734, *T*_max_ = 0.827	*l* = −24→26
14882 measured reflections	

### Refinement

**Table:**

Refinement on *F*^2^	Primary atom site location: structure-invariant direct methods
Least-squares matrix: full	Secondary atom site location: difference Fourier map
*R*[*F*^2^ > 2σ(*F*^2^)] = 0.047	Hydrogen site location: inferred from neighbouring sites
*wR*(*F*^2^) = 0.126	H-atom parameters constrained
*S* = 0.98	*w* = 1/[σ^2^(*F*_o_^2^) + (0.06*P*)^2^] where *P* = (*F*_o_^2^ + 2*F*_c_^2^)/3
10003 reflections	(Δ/σ)_max_ < 0.001
608 parameters	Δρ_max_ = 0.60 e Å^−^^3^
0 restraints	Δρ_min_ = −0.59 e Å^−^^3^

### Special details

**Table:**

Geometry. All esds (except the esd in the dihedral angle between two l.s. planes) are estimated using the full covariance matrix. The cell esds are taken into account individually in the estimation of esds in distances, angles and torsion angles; correlations between esds in cell parameters are only used when they are defined by crystal symmetry. An approximate (isotropic) treatment of cell esds is used for estimating esds involving l.s. planes.
Refinement. Refinement of F^2^ against ALL reflections. The weighted R-factor wR and goodness of fit S are based on F^2^, conventional R-factors R are based on F, with F set to zero for negative F^2^. The threshold expression of F^2^ > 2sigma(F^2^) is used only for calculating R-factors(gt) etc. and is not relevant to the choice of reflections for refinement. R-factors based on F^2^ are statistically about twice as large as those based on F, and R- factors based on ALL data will be even larger.

### Fractional atomic coordinates and isotropic or equivalent isotropic displacement parameters (Å^2^)

**Table:**

	*x*	*y*	*z*	*U*_iso_*/*U*_eq_	Occ. (<1)
Pd1	0.72416 (5)	0.71604 (4)	0.45564 (2)	0.04002 (13)	
Co1	0.45423 (7)	0.73312 (6)	0.26318 (3)	0.03195 (17)	
P1	0.76022 (14)	0.84931 (11)	0.38561 (6)	0.0323 (3)	
C1	0.3718 (7)	0.8474 (6)	0.2772 (3)	0.0563 (17)	
H1	0.4224	0.9308	0.2840	0.068*	
Pd2	0.53088 (5)	0.78107 (4)	0.06221 (2)	0.04233 (13)	
P2	0.51015 (14)	0.63486 (11)	0.12431 (6)	0.0331 (3)	
C2	0.2990 (6)	0.7701 (6)	0.2175 (3)	0.0592 (18)	
H2	0.2927	0.7934	0.1779	0.071*	
C3	0.2363 (6)	0.6497 (6)	0.2280 (3)	0.0530 (15)	
H3	0.1843	0.5807	0.1963	0.064*	
C4	0.2667 (5)	0.6531 (5)	0.2947 (3)	0.0430 (13)	
C5	0.3551 (6)	0.7764 (5)	0.3256 (3)	0.0476 (14)	
H5	0.3946	0.8050	0.3694	0.057*	
C6	0.6348 (5)	0.8196 (4)	0.2430 (2)	0.0310 (11)	
C7	0.6545 (5)	0.7806 (4)	0.3037 (2)	0.0301 (11)	
C8	0.5800 (5)	0.6533 (4)	0.2708 (2)	0.0315 (11)	
C9	0.5603 (5)	0.6945 (4)	0.2091 (2)	0.0313 (11)	
C10	0.6979 (6)	0.9356 (4)	0.2197 (2)	0.0351 (12)	
C11	0.8424 (6)	0.9924 (5)	0.2267 (3)	0.0501 (15)	
H11	0.8976	0.9571	0.2448	0.060*	
C12	0.9045 (7)	1.1006 (6)	0.2071 (3)	0.0649 (19)	
H12	1.0014	1.1380	0.2124	0.078*	
C13	0.8247 (8)	1.1533 (5)	0.1798 (3)	0.071 (2)	
H13	0.8669	1.2257	0.1662	0.085*	
C14	0.6823 (7)	1.0987 (5)	0.1727 (3)	0.0618 (18)	
H14	0.6279	1.1350	0.1551	0.074*	
C15	0.6191 (6)	0.9894 (5)	0.1917 (3)	0.0481 (14)	
H15	0.5220	0.9518	0.1855	0.058*	
C16	0.5674 (5)	0.5406 (4)	0.2916 (2)	0.0329 (11)	
C17	0.5024 (6)	0.5014 (5)	0.3394 (3)	0.0457 (14)	
H17	0.4671	0.5471	0.3587	0.055*	
C18	0.4891 (7)	0.3958 (6)	0.3589 (3)	0.0638 (18)	
H18	0.4443	0.3701	0.3907	0.077*	
C19	0.5425 (8)	0.3279 (6)	0.3310 (4)	0.070 (2)	
H19	0.5308	0.2553	0.3431	0.084*	
C20	0.6126 (8)	0.3672 (6)	0.2853 (3)	0.072 (2)	
H20	0.6516	0.3230	0.2677	0.086*	
C21	0.9370 (5)	0.9072 (4)	0.3733 (2)	0.0331 (11)	
C22	0.9643 (6)	0.8345 (5)	0.3321 (3)	0.0444 (13)	
H22	0.8929	0.7583	0.3117	0.053*	
C23	1.0946 (6)	0.8727 (5)	0.3208 (3)	0.0526 (15)	
H23	1.1117	0.8222	0.2938	0.063*	
C24	1.1996 (6)	0.9865 (6)	0.3500 (3)	0.0676 (19)	
H24	1.2869	1.0141	0.3413	0.081*	
C25	1.1763 (6)	1.0592 (6)	0.3916 (3)	0.0650 (19)	
H25	1.2480	1.1352	0.4120	0.078*	
C26	1.0448 (6)	1.0188 (5)	0.4032 (3)	0.0496 (15)	
H26	1.0294	1.0681	0.4318	0.060*	
C27	0.7412 (5)	0.9859 (4)	0.4061 (2)	0.0371 (12)	
C28	0.6891 (6)	0.9970 (5)	0.4581 (3)	0.0567 (17)	
H28	0.6639	0.9354	0.4813	0.068*	
C29	0.6744 (7)	1.0980 (6)	0.4754 (4)	0.070 (2)	
H29	0.6399	1.1048	0.5104	0.084*	
C30	0.7105 (7)	1.1895 (6)	0.4411 (4)	0.069 (2)	
H30	0.6979	1.2569	0.4521	0.083*	
C31	0.7647 (8)	1.1807 (6)	0.3910 (3)	0.0651 (18)	
H31	0.7924	1.2436	0.3687	0.078*	
C32	0.7787 (7)	1.0783 (5)	0.3731 (3)	0.0548 (16)	
H32	0.8141	1.0724	0.3383	0.066*	
C33	0.3390 (5)	0.5024 (4)	0.1043 (2)	0.0382 (12)	
C34	0.2326 (6)	0.4928 (5)	0.0537 (3)	0.0612 (17)	
H34	0.2496	0.5536	0.0299	0.073*	
C35	0.0994 (7)	0.3932 (6)	0.0373 (4)	0.078 (2)	
H35	0.0281	0.3888	0.0035	0.093*	
C36	0.0750 (7)	0.3034 (6)	0.0711 (3)	0.0667 (19)	
H36	−0.0130	0.2365	0.0601	0.080*	
C37	0.1786 (7)	0.3106 (6)	0.1209 (3)	0.0675 (19)	
H37	0.1606	0.2489	0.1442	0.081*	
C38	0.3102 (6)	0.4083 (5)	0.1375 (3)	0.0559 (16)	
H38	0.3806	0.4110	0.1713	0.067*	
C39	0.6377 (5)	0.5762 (5)	0.1229 (2)	0.0382 (12)	
C40	0.7772 (6)	0.6478 (5)	0.1557 (3)	0.0477 (14)	
H40	0.8041	0.7249	0.1775	0.057*	
C41	0.8782 (6)	0.6077 (6)	0.1572 (3)	0.0610 (17)	
H41	0.9717	0.6577	0.1795	0.073*	
C42	0.8399 (8)	0.4940 (7)	0.1257 (3)	0.070 (2)	
H42	0.9069	0.4660	0.1276	0.084*	
C43	0.7029 (8)	0.4219 (6)	0.0914 (3)	0.070 (2)	
H43	0.6773	0.3456	0.0692	0.084*	
C44	0.6018 (7)	0.4626 (5)	0.0898 (3)	0.0535 (15)	
H44	0.5090	0.4132	0.0664	0.064*	
C52	0.2038 (6)	0.5526 (5)	0.3285 (3)	0.0533 (15)	
H52	0.2789	0.5580	0.3645	0.064*	
C53	0.6248 (7)	0.4743 (5)	0.2656 (3)	0.0511 (15)	
H53	0.6721	0.5012	0.2347	0.061*	
C54	0.0937 (8)	0.5682 (8)	0.3559 (4)	0.101 (3)	
H54A	0.1362	0.6455	0.3837	0.151*	
H54B	0.0567	0.5060	0.3797	0.151*	
H54C	0.0186	0.5630	0.3215	0.151*	
C55	0.1415 (8)	0.4290 (6)	0.2856 (4)	0.089 (2)	
H55A	0.0682	0.4218	0.2496	0.133*	
H55B	0.1031	0.3674	0.3095	0.133*	
H55C	0.2141	0.4200	0.2705	0.133*	
Cl1	0.51400 (16)	0.70395 (13)	0.47871 (7)	0.0526 (4)	
Cl2	0.31495 (17)	0.79401 (14)	0.05176 (8)	0.0607 (4)	
C48	0.7290 (7)	0.8105 (6)	0.0497 (3)	0.073 (2)	
H48A	0.6757	0.7633	0.0768	0.087*	
H48B	0.8215	0.8157	0.0525	0.087*	
C47	0.7535 (9)	0.5996 (7)	0.5217 (4)	0.090 (3)	
H47A	0.6787	0.6237	0.5140	0.107*	
H47B	0.7554	0.5520	0.5546	0.107*	
C49	0.6587 (12)	0.8395 (9)	−0.0045 (5)	0.042 (3)	0.55
H49A	0.7139	0.8581	−0.0358	0.050*	0.55
C49'	0.697 (3)	0.886 (2)	0.0259 (12)	0.105 (9)*	0.45
H49B	0.7839	0.9417	0.0169	0.126*	0.45
C46	0.8156 (14)	0.5940 (10)	0.4751 (6)	0.052 (3)	0.55
H46	0.8562	0.5379	0.4815	0.063*	0.55
C46'	0.862 (2)	0.6582 (17)	0.5070 (10)	0.078 (5)*	0.45
H46'	0.9363	0.6459	0.5348	0.093*	0.45
C45	0.9043 (8)	0.6902 (7)	0.4564 (4)	0.082 (2)	
H45A	0.9942	0.6959	0.4530	0.099*	
H45B	0.8674	0.7363	0.4315	0.099*	
C50	0.6146 (9)	0.9242 (7)	0.0062 (4)	0.092 (3)	
H50A	0.6436	0.9933	−0.0145	0.110*	
H50B	0.5329	0.9045	0.0226	0.110*	
Cl4'	1.3448 (8)	1.1123 (8)	0.1784 (6)	0.137 (3)	0.50
Cl3'	1.0857 (6)	0.9245 (8)	0.1500 (5)	0.175 (3)	0.50
Cl3	1.0708 (6)	0.9902 (5)	0.0675 (4)	0.160 (3)	0.50
Cl4	1.276 (3)	1.067 (2)	0.1853 (10)	0.376 (15)	0.50
C56	1.2044 (18)	0.9887 (15)	0.1210 (8)	0.181 (7)	
H56A	1.1709	1.0175	0.0841	0.217*	
H56B	1.2380	0.9332	0.1066	0.217*	

### Atomic displacement parameters (Å^2^)

**Table:**

	*U*^11^	*U*^22^	*U*^33^	*U*^12^	*U*^13^	*U*^23^
Pd1	0.0423 (3)	0.0414 (2)	0.0371 (2)	0.0195 (2)	0.01012 (19)	0.00980 (18)
Co1	0.0251 (3)	0.0379 (4)	0.0374 (4)	0.0164 (3)	0.0113 (3)	0.0112 (3)
P1	0.0309 (7)	0.0332 (7)	0.0332 (7)	0.0155 (6)	0.0088 (6)	0.0039 (6)
C1	0.050 (4)	0.052 (4)	0.092 (5)	0.034 (3)	0.041 (4)	0.028 (4)
Pd2	0.0447 (3)	0.0415 (3)	0.0407 (3)	0.0177 (2)	0.0134 (2)	0.01344 (19)
P2	0.0294 (7)	0.0344 (7)	0.0332 (7)	0.0123 (6)	0.0085 (6)	0.0065 (6)
C2	0.038 (3)	0.098 (5)	0.069 (5)	0.047 (4)	0.025 (3)	0.043 (4)
C3	0.024 (3)	0.072 (4)	0.056 (4)	0.016 (3)	0.011 (3)	0.010 (3)
C4	0.026 (3)	0.056 (4)	0.052 (4)	0.019 (3)	0.019 (3)	0.016 (3)
C5	0.044 (3)	0.060 (4)	0.053 (4)	0.031 (3)	0.026 (3)	0.014 (3)
C6	0.028 (3)	0.031 (3)	0.038 (3)	0.016 (2)	0.011 (2)	0.006 (2)
C7	0.026 (3)	0.031 (3)	0.037 (3)	0.015 (2)	0.012 (2)	0.007 (2)
C8	0.027 (3)	0.036 (3)	0.034 (3)	0.016 (2)	0.009 (2)	0.005 (2)
C9	0.025 (3)	0.037 (3)	0.032 (3)	0.013 (2)	0.010 (2)	0.006 (2)
C10	0.042 (3)	0.029 (3)	0.032 (3)	0.013 (2)	0.012 (2)	0.004 (2)
C11	0.038 (3)	0.048 (3)	0.055 (4)	0.010 (3)	0.014 (3)	0.013 (3)
C12	0.055 (4)	0.056 (4)	0.066 (4)	0.003 (4)	0.026 (4)	0.024 (3)
C13	0.078 (5)	0.037 (3)	0.081 (5)	0.007 (4)	0.027 (4)	0.024 (3)
C14	0.070 (5)	0.048 (4)	0.065 (4)	0.024 (4)	0.014 (4)	0.022 (3)
C15	0.051 (4)	0.043 (3)	0.055 (4)	0.022 (3)	0.019 (3)	0.020 (3)
C16	0.030 (3)	0.034 (3)	0.031 (3)	0.014 (2)	0.000 (2)	0.004 (2)
C17	0.041 (3)	0.053 (3)	0.050 (3)	0.026 (3)	0.014 (3)	0.020 (3)
C18	0.062 (4)	0.066 (4)	0.067 (4)	0.025 (4)	0.024 (4)	0.032 (4)
C19	0.081 (5)	0.044 (4)	0.083 (5)	0.032 (4)	0.008 (4)	0.023 (4)
C20	0.105 (6)	0.061 (4)	0.078 (5)	0.060 (5)	0.028 (5)	0.022 (4)
C21	0.029 (3)	0.036 (3)	0.034 (3)	0.018 (2)	0.003 (2)	0.003 (2)
C22	0.031 (3)	0.045 (3)	0.057 (4)	0.019 (3)	0.011 (3)	0.005 (3)
C23	0.039 (3)	0.059 (4)	0.059 (4)	0.024 (3)	0.013 (3)	−0.003 (3)
C24	0.030 (3)	0.087 (5)	0.079 (5)	0.021 (4)	0.018 (3)	0.003 (4)
C25	0.036 (3)	0.053 (4)	0.082 (5)	0.003 (3)	0.015 (3)	−0.011 (3)
C26	0.033 (3)	0.051 (4)	0.053 (4)	0.013 (3)	0.008 (3)	−0.005 (3)
C27	0.039 (3)	0.039 (3)	0.037 (3)	0.023 (3)	0.007 (2)	0.003 (2)
C28	0.062 (4)	0.042 (3)	0.072 (4)	0.022 (3)	0.038 (4)	0.004 (3)
C29	0.074 (5)	0.064 (4)	0.085 (5)	0.036 (4)	0.041 (4)	0.000 (4)
C30	0.075 (5)	0.053 (4)	0.087 (5)	0.046 (4)	0.007 (4)	−0.006 (4)
C31	0.087 (5)	0.056 (4)	0.065 (4)	0.046 (4)	0.014 (4)	0.017 (3)
C32	0.077 (5)	0.053 (4)	0.044 (3)	0.040 (4)	0.014 (3)	0.004 (3)
C33	0.035 (3)	0.034 (3)	0.038 (3)	0.008 (2)	0.012 (2)	0.001 (2)
C34	0.043 (4)	0.049 (4)	0.071 (4)	0.010 (3)	0.000 (3)	0.013 (3)
C35	0.047 (4)	0.062 (4)	0.084 (5)	0.005 (4)	−0.017 (4)	0.008 (4)
C36	0.039 (4)	0.052 (4)	0.082 (5)	0.002 (3)	0.009 (4)	0.002 (4)
C37	0.058 (4)	0.053 (4)	0.071 (5)	0.003 (4)	0.021 (4)	0.020 (3)
C38	0.047 (4)	0.053 (4)	0.057 (4)	0.015 (3)	0.006 (3)	0.017 (3)
C39	0.036 (3)	0.040 (3)	0.038 (3)	0.015 (3)	0.014 (2)	0.006 (2)
C40	0.041 (3)	0.054 (4)	0.050 (4)	0.024 (3)	0.014 (3)	0.007 (3)
C41	0.039 (3)	0.077 (5)	0.069 (4)	0.030 (4)	0.014 (3)	0.002 (4)
C42	0.067 (5)	0.101 (6)	0.065 (5)	0.061 (5)	0.017 (4)	0.004 (4)
C43	0.080 (5)	0.072 (5)	0.071 (5)	0.051 (4)	0.015 (4)	−0.008 (4)
C44	0.050 (4)	0.058 (4)	0.049 (4)	0.027 (3)	0.003 (3)	0.000 (3)
C52	0.038 (3)	0.061 (4)	0.062 (4)	0.019 (3)	0.020 (3)	0.025 (3)
C53	0.068 (4)	0.056 (4)	0.046 (3)	0.039 (3)	0.023 (3)	0.018 (3)
C54	0.079 (6)	0.121 (7)	0.146 (8)	0.051 (5)	0.085 (6)	0.080 (6)
C55	0.077 (6)	0.067 (5)	0.097 (6)	0.009 (4)	0.021 (5)	0.024 (4)
Cl1	0.0488 (9)	0.0544 (9)	0.0561 (9)	0.0202 (8)	0.0234 (7)	0.0133 (7)
Cl2	0.0568 (10)	0.0636 (10)	0.0678 (10)	0.0341 (9)	0.0130 (8)	0.0171 (8)
C48	0.042 (4)	0.103 (6)	0.096 (6)	0.033 (4)	0.049 (4)	0.060 (5)
C47	0.096 (6)	0.110 (6)	0.110 (7)	0.074 (6)	0.042 (5)	0.079 (5)
C49	0.050 (7)	0.036 (6)	0.037 (6)	0.006 (5)	0.035 (5)	0.012 (5)
C46	0.073 (8)	0.045 (7)	0.053 (7)	0.045 (7)	0.004 (7)	0.015 (6)
C45	0.077 (5)	0.113 (6)	0.111 (6)	0.078 (5)	0.042 (5)	0.065 (5)
C50	0.099 (6)	0.098 (6)	0.120 (7)	0.049 (5)	0.081 (6)	0.082 (6)
Cl4'	0.096 (4)	0.098 (4)	0.166 (8)	0.042 (3)	−0.037 (4)	−0.031 (4)
Cl3'	0.082 (4)	0.233 (8)	0.229 (8)	0.060 (5)	0.078 (5)	0.115 (7)
Cl3	0.067 (3)	0.119 (4)	0.253 (8)	0.008 (3)	0.058 (4)	−0.015 (5)
Cl4	0.64 (4)	0.56 (3)	0.214 (15)	0.53 (3)	0.08 (2)	0.11 (2)
C56	0.239 (18)	0.266 (18)	0.162 (14)	0.200 (16)	0.111 (14)	0.064 (13)

### Geometric parameters (Å, °)

**Table:**

Pd1—C46'	2.09 (2)	C25—H25	0.9300
Pd1—C45	2.116 (6)	C26—H26	0.9300
Pd1—C46	2.171 (10)	C27—C32	1.368 (7)
Pd1—C47	2.195 (6)	C27—C28	1.389 (7)
Pd1—P1	2.3132 (15)	C28—C29	1.372 (8)
Pd1—Cl1	2.3901 (17)	C28—H28	0.9300
Co1—C6	1.964 (5)	C29—C30	1.378 (9)
Co1—C7	1.973 (5)	C29—H29	0.9300
Co1—C9	1.976 (5)	C30—C31	1.361 (9)
Co1—C8	1.998 (5)	C30—H30	0.9300
Co1—C1	2.018 (5)	C31—C32	1.387 (8)
Co1—C2	2.022 (5)	C31—H31	0.9300
Co1—C5	2.072 (5)	C32—H32	0.9300
Co1—C3	2.075 (5)	C33—C34	1.376 (8)
Co1—C4	2.146 (5)	C33—C38	1.386 (7)
P1—C7	1.813 (5)	C34—C35	1.399 (8)
P1—C21	1.835 (5)	C34—H34	0.9300
P1—C27	1.837 (5)	C35—C36	1.354 (9)
C1—C2	1.400 (9)	C35—H35	0.9300
C1—C5	1.421 (8)	C36—C37	1.355 (9)
C1—H1	0.9300	C36—H36	0.9300
Pd2—C48	2.110 (6)	C37—C38	1.378 (8)
Pd2—C49'	2.09 (3)	C37—H37	0.9300
Pd2—C49	2.174 (9)	C38—H38	0.9300
Pd2—C50	2.223 (6)	C39—C40	1.380 (7)
Pd2—P2	2.3215 (15)	C39—C44	1.389 (7)
Pd2—Cl2	2.3897 (18)	C40—C41	1.384 (8)
P2—C9	1.806 (5)	C40—H40	0.9300
P2—C33	1.823 (5)	C41—C42	1.372 (9)
P2—C39	1.830 (5)	C41—H41	0.9300
C2—C3	1.424 (8)	C42—C43	1.369 (9)
C2—H2	0.9300	C42—H42	0.9300
C3—C4	1.412 (8)	C43—C44	1.391 (8)
C3—H3	0.9300	C43—H43	0.9300
C4—C5	1.433 (8)	C44—H44	0.9300
C4—C52	1.489 (7)	C52—C54	1.520 (8)
C5—H5	0.9300	C52—C55	1.526 (9)
C6—C7	1.449 (6)	C52—H52	0.9800
C6—C9	1.460 (6)	C53—H53	0.9300
C6—C10	1.500 (6)	C54—H54A	0.9600
C7—C8	1.475 (6)	C54—H54B	0.9600
C8—C16	1.473 (7)	C54—H54C	0.9600
C8—C9	1.482 (6)	C55—H55A	0.9600
C10—C15	1.377 (7)	C55—H55B	0.9600
C10—C11	1.390 (7)	C55—H55C	0.9600
C11—C12	1.381 (8)	C48—C49'	1.25 (3)
C11—H11	0.9300	C48—C49	1.425 (14)
C12—C13	1.369 (9)	C48—H48A	0.9700
C12—H12	0.9300	C48—H48B	0.9700
C13—C14	1.370 (9)	C47—C46'	1.222 (19)
C13—H13	0.9300	C47—C46	1.344 (14)
C14—C15	1.386 (7)	C47—H47A	0.9700
C14—H14	0.9300	C47—H47B	0.9700
C15—H15	0.9300	C49—C49'	0.75 (2)
C16—C53	1.382 (7)	C49—C50	1.358 (13)
C16—C17	1.386 (7)	C49—H49A	0.9800
C17—C18	1.377 (8)	C49'—C50	1.21 (3)
C17—H17	0.9300	C49'—H49B	0.9800
C18—C19	1.382 (9)	C46—C46'	0.893 (18)
C18—H18	0.9300	C46—C45	1.342 (13)
C19—C20	1.374 (9)	C46—H46	0.9800
C19—H19	0.9300	C46'—C45	1.31 (2)
C20—C53	1.400 (8)	C46'—H46'	0.9800
C20—H20	0.9300	C45—H45A	0.9700
C21—C26	1.374 (7)	C45—H45B	0.9700
C21—C22	1.387 (7)	C50—H50A	0.9700
C22—C23	1.375 (7)	C50—H50B	0.9700
C22—H22	0.9300	Cl4'—C56	1.78 (2)
C23—C24	1.379 (8)	Cl3'—C56	1.502 (14)
C23—H23	0.9300	Cl3—C56	1.670 (17)
C24—C25	1.367 (8)	Cl4—C56	1.48 (2)
C24—H24	0.9300	C56—H56A	0.9700
C25—C26	1.387 (8)	C56—H56B	0.9700
			
C46'—Pd1—C45	36.2 (5)	C18—C17—C16	121.1 (6)
C46'—Pd1—C46	24.1 (5)	C18—C17—H17	119.5
C45—Pd1—C46	36.5 (4)	C16—C17—H17	119.5
C46'—Pd1—C47	33.0 (5)	C17—C18—C19	119.8 (6)
C45—Pd1—C47	66.9 (3)	C17—C18—H18	120.1
C46—Pd1—C47	35.9 (4)	C19—C18—H18	120.1
C46'—Pd1—P1	130.0 (5)	C20—C19—C18	120.4 (6)
C45—Pd1—P1	96.36 (19)	C20—C19—H19	119.8
C46—Pd1—P1	129.6 (4)	C18—C19—H19	119.8
C47—Pd1—P1	163.0 (2)	C19—C20—C53	119.3 (6)
C46'—Pd1—Cl1	126.2 (6)	C19—C20—H20	120.3
C45—Pd1—Cl1	161.9 (2)	C53—C20—H20	120.3
C46—Pd1—Cl1	126.6 (4)	C26—C21—C22	118.1 (5)
C47—Pd1—Cl1	94.9 (2)	C26—C21—P1	123.4 (4)
P1—Pd1—Cl1	101.77 (5)	C22—C21—P1	118.6 (4)
C6—Co1—C7	43.20 (19)	C23—C22—C21	121.4 (5)
C6—Co1—C9	43.51 (19)	C23—C22—H22	119.3
C7—Co1—C9	63.05 (19)	C21—C22—H22	119.3
C6—Co1—C8	63.45 (19)	C22—C23—C24	119.3 (5)
C7—Co1—C8	43.60 (19)	C22—C23—H23	120.3
C9—Co1—C8	43.78 (19)	C24—C23—H23	120.3
C6—Co1—C1	109.4 (2)	C25—C24—C23	120.4 (6)
C7—Co1—C1	120.6 (2)	C25—C24—H24	119.8
C9—Co1—C1	141.4 (2)	C23—C24—H24	119.8
C8—Co1—C1	163.6 (3)	C24—C25—C26	119.6 (6)
C6—Co1—C2	114.4 (2)	C24—C25—H25	120.2
C7—Co1—C2	151.5 (2)	C26—C25—H25	120.2
C9—Co1—C2	116.0 (2)	C21—C26—C25	121.2 (5)
C8—Co1—C2	155.2 (3)	C21—C26—H26	119.4
C1—Co1—C2	40.5 (3)	C25—C26—H26	119.4
C6—Co1—C5	134.4 (2)	C32—C27—C28	118.5 (5)
C7—Co1—C5	112.9 (2)	C32—C27—P1	122.7 (4)
C9—Co1—C5	175.9 (2)	C28—C27—P1	118.7 (4)
C8—Co1—C5	132.9 (2)	C29—C28—C27	120.6 (6)
C1—Co1—C5	40.6 (2)	C29—C28—H28	119.7
C2—Co1—C5	67.9 (2)	C27—C28—H28	119.7
C6—Co1—C3	145.7 (2)	C28—C29—C30	120.2 (6)
C7—Co1—C3	167.8 (2)	C28—C29—H29	119.9
C9—Co1—C3	116.7 (2)	C30—C29—H29	119.9
C8—Co1—C3	126.7 (2)	C31—C30—C29	119.6 (6)
C1—Co1—C3	68.0 (3)	C31—C30—H30	120.2
C2—Co1—C3	40.7 (2)	C29—C30—H30	120.2
C5—Co1—C3	67.1 (2)	C30—C31—C32	120.2 (6)
C6—Co1—C4	173.7 (2)	C30—C31—H31	119.9
C7—Co1—C4	133.2 (2)	C32—C31—H31	119.9
C9—Co1—C4	142.2 (2)	C27—C32—C31	120.8 (6)
C8—Co1—C4	118.2 (2)	C27—C32—H32	119.6
C1—Co1—C4	67.2 (2)	C31—C32—H32	119.6
C2—Co1—C4	66.9 (2)	C34—C33—C38	117.5 (5)
C5—Co1—C4	39.7 (2)	C34—C33—P2	119.6 (4)
C3—Co1—C4	39.0 (2)	C38—C33—P2	123.0 (5)
C7—P1—C21	99.7 (2)	C33—C34—C35	121.2 (6)
C7—P1—C27	106.7 (2)	C33—C34—H34	119.4
C21—P1—C27	103.7 (2)	C35—C34—H34	119.4
C7—P1—Pd1	113.91 (16)	C36—C35—C34	119.6 (7)
C21—P1—Pd1	112.87 (16)	C36—C35—H35	120.2
C27—P1—Pd1	117.94 (17)	C34—C35—H35	120.2
C2—C1—C5	108.2 (6)	C37—C36—C35	120.2 (6)
C2—C1—Co1	69.9 (3)	C37—C36—H36	119.9
C5—C1—Co1	71.7 (3)	C35—C36—H36	119.9
C2—C1—H1	125.9	C36—C37—C38	120.8 (6)
C5—C1—H1	125.9	C36—C37—H37	119.6
Co1—C1—H1	124.2	C38—C37—H37	119.6
C48—Pd2—C49'	34.6 (7)	C37—C38—C33	120.7 (6)
C48—Pd2—C49	38.8 (4)	C37—C38—H38	119.6
C49'—Pd2—C49	20.1 (6)	C33—C38—H38	119.6
C48—Pd2—C50	66.8 (3)	C40—C39—C44	117.7 (5)
C49'—Pd2—C50	32.3 (7)	C40—C39—P2	119.0 (4)
C49—Pd2—C50	36.0 (3)	C44—C39—P2	123.4 (4)
C48—Pd2—P2	96.09 (19)	C39—C40—C41	121.6 (6)
C49'—Pd2—P2	130.5 (8)	C39—C40—H40	119.2
C49—Pd2—P2	129.5 (3)	C41—C40—H40	119.2
C50—Pd2—P2	162.7 (2)	C42—C41—C40	119.8 (6)
C48—Pd2—Cl2	160.51 (19)	C42—C41—H41	120.1
C49'—Pd2—Cl2	126.2 (8)	C40—C41—H41	120.1
C49—Pd2—Cl2	122.7 (3)	C43—C42—C41	120.0 (6)
C50—Pd2—Cl2	93.9 (2)	C43—C42—H42	120.0
P2—Pd2—Cl2	103.31 (5)	C41—C42—H42	120.0
C9—P2—C33	108.3 (2)	C42—C43—C44	120.1 (6)
C9—P2—C39	99.4 (2)	C42—C43—H43	120.0
C33—P2—C39	105.3 (2)	C44—C43—H43	120.0
C9—P2—Pd2	112.77 (16)	C39—C44—C43	120.9 (6)
C33—P2—Pd2	116.33 (18)	C39—C44—H44	119.6
C39—P2—Pd2	113.17 (17)	C43—C44—H44	119.6
C1—C2—C3	108.2 (6)	C4—C52—C54	109.3 (5)
C1—C2—Co1	69.6 (3)	C4—C52—C55	112.6 (5)
C3—C2—Co1	71.7 (3)	C54—C52—C55	111.2 (6)
C1—C2—H2	125.9	C4—C52—H52	107.9
C3—C2—H2	125.9	C54—C52—H52	107.9
Co1—C2—H2	124.5	C55—C52—H52	107.9
C4—C3—C2	108.3 (6)	C16—C53—C20	120.8 (6)
C4—C3—Co1	73.2 (3)	C16—C53—H53	119.6
C2—C3—Co1	67.7 (3)	C20—C53—H53	119.6
C4—C3—H3	125.8	C52—C54—H54A	109.5
C2—C3—H3	125.8	C52—C54—H54B	109.5
Co1—C3—H3	124.9	H54A—C54—H54B	109.5
C3—C4—C5	107.2 (5)	C52—C54—H54C	109.5
C3—C4—C52	127.5 (5)	H54A—C54—H54C	109.5
C5—C4—C52	124.7 (5)	H54B—C54—H54C	109.5
C3—C4—Co1	67.8 (3)	C52—C55—H55A	109.5
C5—C4—Co1	67.4 (3)	C52—C55—H55B	109.5
C52—C4—Co1	136.6 (4)	H55A—C55—H55B	109.5
C1—C5—C4	107.8 (5)	C52—C55—H55C	109.5
C1—C5—Co1	67.7 (3)	H55A—C55—H55C	109.5
C4—C5—Co1	72.9 (3)	H55B—C55—H55C	109.5
C1—C5—H5	126.1	C49—C48—Pd2	73.0 (5)
C4—C5—H5	126.1	C49—C48—H48A	119.8
Co1—C5—H5	124.9	Pd2—C48—H48A	49.5
C7—C6—C9	90.4 (4)	C49—C48—H48B	119.8
C7—C6—C10	135.9 (4)	Pd2—C48—H48B	167.0
C9—C6—C10	131.5 (4)	H48A—C48—H48B	117.7
C7—C6—Co1	68.7 (3)	C46—C47—Pd1	71.1 (5)
C9—C6—Co1	68.7 (3)	C46—C47—H47A	119.4
C10—C6—Co1	132.3 (3)	Pd1—C47—H47A	53.1
C6—C7—C8	90.9 (4)	C46—C47—H47B	119.4
C6—C7—P1	136.9 (4)	Pd1—C47—H47B	169.5
C8—C7—P1	130.8 (4)	H47A—C47—H47B	117.0
C6—C7—Co1	68.1 (3)	C50—C49—C48	117.9 (9)
C8—C7—Co1	69.1 (3)	C50—C49—Pd2	74.0 (5)
P1—C7—Co1	130.6 (2)	C48—C49—Pd2	68.1 (4)
C16—C8—C7	133.0 (4)	C50—C49—H49A	110.6
C16—C8—C9	136.7 (4)	C48—C49—H49A	110.6
C7—C8—C9	88.6 (4)	Pd2—C49—H49A	174.8
C16—C8—Co1	132.3 (3)	C47—C46—C45	124.6 (10)
C7—C8—Co1	67.3 (3)	C47—C46—Pd1	73.1 (5)
C9—C8—Co1	67.3 (3)	C45—C46—Pd1	69.6 (5)
C6—C9—C8	90.2 (4)	C47—C46—H46	109.4
C6—C9—P2	129.4 (4)	C45—C46—H46	109.4
C8—C9—P2	138.9 (4)	Pd1—C46—H46	177.1
C6—C9—Co1	67.8 (3)	Pd1—C46'—H46'	169.9
C8—C9—Co1	68.9 (3)	C46—C45—Pd1	74.0 (5)
P2—C9—Co1	130.6 (3)	C46—C45—H45A	119.4
C15—C10—C11	118.1 (5)	Pd1—C45—H45A	166.6
C15—C10—C6	122.9 (5)	C46—C45—H45B	119.4
C11—C10—C6	119.0 (5)	Pd1—C45—H45B	50.4
C12—C11—C10	120.6 (6)	H45A—C45—H45B	117.1
C12—C11—H11	119.7	C49—C50—Pd2	70.0 (5)
C10—C11—H11	119.7	C49—C50—H50A	119.8
C13—C12—C11	120.5 (6)	Pd2—C50—H50A	170.2
C13—C12—H12	119.7	C49—C50—H50B	119.8
C11—C12—H12	119.7	Pd2—C50—H50B	53.5
C12—C13—C14	119.6 (6)	H50A—C50—H50B	117.6
C12—C13—H13	120.2	Cl4—C56—Cl3	125.4 (11)
C14—C13—H13	120.2	Cl3'—C56—Cl4'	110.1 (11)
C13—C14—C15	120.1 (6)	Cl4—C56—H56A	121.9
C13—C14—H14	120.0	Cl3'—C56—H56A	109.6
C15—C14—H14	120.0	Cl4'—C56—H56A	109.6
C10—C15—C14	121.1 (6)	Cl4—C56—H56B	119.6
C10—C15—H15	119.5	Cl3'—C56—H56B	109.6
C14—C15—H15	119.5	Cl3—C56—H56B	115.0
C53—C16—C17	118.6 (5)	Cl4'—C56—H56B	109.6
C53—C16—C8	120.8 (5)	H56A—C56—H56B	108.2
C17—C16—C8	120.6 (5)		
